# Association Between Air Pollution and Lung Lobar Emphysema in COPD

**DOI:** 10.3389/fmed.2021.705792

**Published:** 2021-09-21

**Authors:** Nguyen Thanh Tung, Shu-Chuan Ho, Yueh-Hsun Lu, Tzu-Tao Chen, Kang-Yun Lee, Kuan-Yuan Chen, Chih-Da Wu, Kian Fan Chung, Han-Pin Kuo, Huynh Nguyen Xuan Thao, Hoang Ba Dung, Tran Phan Chung Thuy, Sheng-Ming Wu, Hsiao-Yun Kou, Yueh-Lun Lee, Hsiao-Chi Chuang

**Affiliations:** ^1^International Ph.D. Program in Medicine, College of Medicine, Taipei Medical University, Taipei, Taiwan; ^2^Otorhinolaryngology Department, Cho Ray Hospital, Ho Chi Minh City, Vietnam; ^3^School of Respiratory Therapy, College of Medicine, Taipei Medical University, Taipei, Taiwan; ^4^Department of Radiology, Shuang Ho Hospital, Taipei Medical University, New Taipei City, Taiwan; ^5^Department of Radiology, School of Medicine, College of Medicine, Taipei Medical University, Taipei, Taiwan; ^6^Division of Pulmonary Medicine, Department of Internal Medicine, Shuang Ho Hospital, Taipei Medical University, New Taipei City, Taiwan; ^7^Division of Pulmonary Medicine, Department of Internal Medicine, School of Medicine, College of Medicine, Taipei Medical University, Taipei, Taiwan; ^8^Department of Geomatics, National Cheng Kung University, Tainan City, Taiwan; ^9^National Institute of Environmental Health Sciences, National Health Research Institutes, Miaoli, Taiwan; ^10^National Heart and Lung Institute, Imperial College London, London, United Kingdom; ^11^Otorhinolaryngology Department, Ho Chi Minh City University of Medicine and Pharmacy, Ho Chi Minh City, Vietnam; ^12^Otorhinolaryngology Department, Faculty of Medicine, Vietnam National University Ho Chi Minh City, Ho Chi Minh City, Vietnam; ^13^Department of Microbiology and Immunology, School of Medicine, College of Medicine, Taipei Medical University, Taipei, Taiwan; ^14^Cell Physiology and Molecular Image Research Center, Wan Fang Hospital, Taipei Medical University, Taipei, Taiwan

**Keywords:** air pollution, BODE, computed tomography, COPD, LAA

## Abstract

The development of emphysema has been linked to air pollution; however, the association of air pollution with the extent of lobar emphysema remains unclear. This study examined the association of particulate matter <2.5 μm in aerodynamic diameters (PM_2.5_) (≤2.5 μm), nitrogen dioxide (NO_2_), and ozone (O_3_) level of exposure with the presence of emphysema in 86 patients with chronic obstructive pulmonary disease (COPD). Exposure to the air pollution estimated using the land-use regression model was associated with lung function, BODE (a body mass index, degree of obstruction, dyspnea severity, and exercise capacity index) quartiles, and emphysema measured as low-attenuation areas on high-resolution CT (HR-CT) lung scans. Using paraseptal emphysema as the reference group, we observed that a 1 ppb increase in O_3_ was associated with a 1.798-fold increased crude odds ratio of panlobular emphysema (*p* < 0.05). We observed that PM_2.5_ was associated with BODE quartiles, modified Medical Research Council (mMRC) dyspnea score, and exercise capacity (all *p* < 0.05). We found that PM_2.5_, NO_2_, and O_3_ were associated with an increased degree of upper lobe emphysema and lower lobe emphysema (all *p* < 0.05). Furthermore, we observed that an increase in PM_2.5_, NO_2_, and O_3_ was associated with greater increases in upper lobe emphysema than in lower lobe emphysema. In conclusion, exposure to O_3_ can be associated with a higher risk of panlobular emphysema than paraseptal emphysema in patients with COPD. Emphysema severity in lung lobes, especially the upper lobes, may be linked to air pollution exposure in COPD.

## Definition

Lobar percent emphysema, the voxel numbers < -950 Hounsfield units (HUs) in a lung lobe field divided by the total voxel numbers in that lung lobe field; centrilobular emphysema, low attenuated areas (LAAs) located within the central portion of the pulmonary lobe; paraseptal emphysema, LAA in the more distal alveoli adjacent to the visceral pleura or interlobular septa; and panlobular emphysema, diffuse LAA that distributes throughout the pulmonary lobe.

## Introduction

Chronic obstructive pulmonary disease (COPD) is an irreversible and progressive respiratory condition. COPD is currently the chronic disease associated with one of the highest mortality in the world, and also ranks fifth worldwide in terms of disease burden (1, 2). In previous studies, air pollution has been associated with increased risk of COPD and reduced lung function (3–6). Specifically, a 5 μg/m^3^ increase in 1-year particulate matter <2.5 μm in aerodynamic diameters (PM_2.5_ concentrations has been associated with 1.52-fold increased odds ratio (OR) of COPD prevalence (95% CI: 1.42–1.62), while a 10 μg/m^3^ increase in 1-year nitrogen dioxide (NO_2_) concentrations was associated with 1.12-fold increased OR of COPD prevalence (95% CI: 1.10–1.14) (7). Therefore, air pollution can be considered as a risk factor for the development of COPD by the Chronic Obstructive Lung Disease (GOLD) guideline (8).

The hybrid kriging/land-use regression (LUR) combined two methods (i.e., kriging interpolation and LUR) to detect small-scale variation in air pollution. This model also takes into consideration the local emission sources (i.e., temples and restaurants) to achieve a more accurate prediction of annual NO_2_ variability (*R*^2^ = 0.90) (9). Another study in Taiwan captured 85% of annual PM_2.5_ variation by using this method (10). Utilizing estimates from the hybrid kriging/LUR, our previous study showed that 0.99 μg/m^3^ increase in PM_2.5_ resulted in 0.011 kg increase in right arm fat mass, whereas 2.45 ppb increase in NO_2_ resulted in 0.181 kg decrease in muscle mass (*p* < 0.05) (11).

Lung emphysema is characterized by the destruction of alveolar septal structures and the loss of lung parenchyma (12). Emphysema is categorized into three major subtypes based on its distribution in secondary lung lobules: centrilobular, paraseptal, and panlobular emphysema (13–15). Centrilobular emphysema has been characterized as low attenuated areas (LAAs) in the central portion of the pulmonary lobe, paraseptal emphysema as LAA in the more distal alveoli adjacent to the visceral pleura or interlobular septa, and panlobular as diffuse LAA that distributes throughout the pulmonary lobe (14, 16, 17). Previous studies have reported an association between smoking and increased risk of centrilobular emphysema (14, 18).

Emphysema can be examined quantitatively on high-resolution CT (HRCT) imaging by measuring the LAA of the lung. The percentage of LAA (or percent emphysema) on HRCT scans has been associated with the decrease in lung function in patients with COPD (19). It was reported that cigarette smoke affected emphysema preferentially in the upper lobes (14, 20). Previous studies have demonstrated the association of air pollution with emphysema severity of the total lung (21, 22). However, the link between exposure to air pollution and the degree of emphysema at the lobular level has not been reported. We hypothesized that exposure to air pollution was associated with the emphysema severity at the lung lobular level. Meanwhile, lung function and BODE index [a composite measure of body-mass index (BMI), degree of obstruction, dyspnea severity, and exercise capacity (23)] were considered clinical outcomes related to the severity of COPD and emphysema. Therefore, we examined the association of 1-year exposure to PM_2.5_, NO_2_, and ozone (O_3_) with the extent of emphysema in different lung lobes of COPD subjects, while also assessing the associations between 1-year exposure to air pollutants with lung function and the degree of incapacity as measured by the BODE index.

## Materials and Methods

### Study Subjects

We conducted a retrospective cross-sectional study in 86 patients with COPD recruited from the COPD clinic of a respiratory department of a hospital in New Taipei, Taiwan. The patients underwent HRCT of the thorax between April 2010 and October 2019. The inclusion criteria in this study were: (1) having been diagnosed with COPD by a post-bronchodilator forced expiratory volume in the first second (FEV_1_)/forced vital capacity (FVC) ratio of <70% (1) and (2) being between 40 years old and 90 years old. The smoking statuses of the patients were collected by oral questionnaire. Patients with a known malignancy, progressive inflammatory condition (i.e., bronchiectasis, asthma, or other non-COPD-related diseases), or exacerbation during the 3 months before the study were excluded. The Ethics Committee of the Taipei Medical University-Joint Institutional Review Board approved this study (Approval No. N202003075).

### Ambient Air Pollution Exposure

Individual-level exposure to air pollutants (PM_2.5_, NO_2_, and O_3_) was predicted by a hybrid kriging/LUR approach, which was previously demonstrated (9, 10). Briefly, mean air pollutant data were obtained from Taiwan Environmental Protection Administration (EPA) air quality monitoring stations (https://airtw.epa.gov.tw/). The Environment Resource Database, Point of Interest, Land-use Investigation of Taiwan, Traffic Network Digital Map, Digital Terrain Model, Industrial Development Bureau Database, and Normalized Difference Vegetation Index were included to build the model. The regression model takes into consideration the traffic intensity, weather, population density, industry emissions, elevation, vegetation distribution, the number of temples, and the number of restaurants to calculate residential air pollution levels. Daily PM_2.5_, NO_2_, and O_3_ levels were then accumulated into 1-year average concentrations. Land-use predictors with a Spearman's correlation coefficient larger than 0.4 with an effect on air pollutants were entered into a stepwise linear regression. Furthermore, to improve the robustness of the LUR model, a set of pollutant levels was created through a leave-one-out kriging interpolation and added to the model. Average individual exposure to air pollution was estimated for 1 year before the HRCT assessment.

### Lung Function

Lung function data were collected from each subject retrospectively from the hospital records of the subject. Spirometry was conducted according to the American Thoracic Society/European Respiratory Society guidelines (24). Lung function tests were performed once right before conducting the HRCT.

### BODE Index

BODE index, namely, BMI, degree of obstruction, dyspnea severity, and exercise capacity, has proven to be a predictor of COPD mortality and severity (23, 25). Previous studies reported that exposure to air pollution could cause adverse effects on dyspnea and exercise capacity (26, 27). A subgroup of four variables (i.e., the BMI scale, airflow obstruction index, modified Medical Research Council (mMRC) dyspnea scale (28), and the exercise capacity index) is included in the BODE index, which is shown in Supplementary Table 1. Airflow obstruction index was defined as the FEV_1_ (% predicted). Exercise capacity index was defined as the distance walked in 6 min (meters). The BODE index was categorized into four quartiles: quartile 1 by a score of 0–2; quartile 2 by a score of 3–4; quartile 3 by a score of 5–6; and quartile 4 by a score of 7–10 as previously described (29). Quartile 1 represents the least severity, while quartile 4 represents the most severity.

### Emphysema Severity

HRCT scans were acquired at suspended full inspiration. APOLLO version 1.2 software (VIDA Diagnostics, Coralville, IA, USA) was employed to assess the image attenuation on full-lung scans at a single reading center by trained readers without the knowledge of the characteristics of participants. The lung volume was calculated, and all voxels in the lung were identified. The percent emphysema (%LAA) on CT scans was determined as the voxel numbers < -950 Hounsfield units in a lung field divided by the total voxel numbers in that lung field based upon pathological comparisons (30, 31). Emphysema severity was categorized into three levels: level 1 if 1% ≤ %LAA < 5%, level 2 if 5% ≤ %LAA < 25%, and level 3 if 25% ≤ %LAA < 50%.

### Statistical Analysis

Tests of normality were used to determine if the data were normally distributed. The extremely low and high values outside percentiles 1 and 99 were replaced by using a winsorization approach to minimize the influence of severe outliers (32). Upper lobe LAA was defined as right upper lobe LAA plus left upper lobe LAA. Lower lobe LAA was defined as right lower lobe LAA plus left lower lobe LAA. We performed a generalized linear model, adjusted for age, sex, BMI, and smoking pack-years, to identify the associations of 1-year air pollution exposure in the five lung lobes with lung function, BODE quartiles, and the percent emphysema in the left upper lobe, left lower lobe, left lung, right upper lobe, right middle lobe, right lower lobe, right lung, upper lobes, lower lobes, and total lung. The beta coefficients (β) were calculated to estimate the contribution of each of the individual variables. Also, the crude OR of outcome variables of predominant centrilobular and panlobular emphysema with the reference group (paraseptal emphysema group) was investigated by a multinomial logistic regression model. To calculate the adjusted OR, we performed the multinomial logistic regression adjusting for age, sex, BMI, and smoking pack-years. SPSS version 22.0.0.0 for Windows statistical software (SPSS Inc., Chicago, IL, USA) was used for data analysis. The value of *p* < 0.05 was set as statistically significant.

## Results

### Characteristics of the Study Subjects

The baseline characteristics of 86 patients enrolled in our study are summarized in Table 1. Overall, the patients had a mean age of 70.4 ± 7.9 years, and 91.9% were men. Their BMI was 23.3 ± 4.4 kg/m^2^. About 40.7% of the subjects were current smokers, 51.2% were ex-smokers, and 8.1% were non-smokers. The average smoking pack-years was 50.4 ± 37.9 years. In terms of lung function, the patients had a mean FEV_1_ (% predicted) of 56.6 ± 19.8%, an average FEV_1_ of 1.3 ± 0.5 L, and FEV_1_/FVC ratio of 52.3 ± 10.0%. Mean outcomes were as follows: BODE quartiles (1.8 ± 1.1 points), mean BMI scale (0.3 ± 0.5 points), mean airflow obstruction index (1.3 ± 1.1 points), mean mMRC dyspnea scale (0.7 ± 0.8 points), and mean exercise capacity index (0.7 ± 1.0 points).

**Table 1:** Demographic characteristics of study subjects.

**Characteristics**	**Mean ± SD**
Total	*N* = 86
Age, years	70.4 ± 7.9
Male, % (*n*)	91.9 (79)
Body mass index, kg/m^2^	23.3 ± 4.4
**Smoking, % (** ***n*** **)**	
Current	40.7 (35)
Ex-smoker	51.2 (44)
Non-smoker	8.1 (7)
**Lung function**	
FEV_1_, %	56.6 ± 19.8
FEV_1_, L	1.3 ± 0.5
FEV_1_/FVC, %	52.3 ± 10.0
BODE quartiles, point	1.8 ± 1.1
BMI scale, point	0.3 ± 0.5
Airflow obstruction index, point	1.3 ± 1.1
mMRC dyspnea scale, point	0.7 ± 0.8
Exercise capacity index, point	0.7 ± 1.0
**Emphysema subtypes**	
Centrilobular, % (*n*)	66.3 (57)
Paraseptal, % (*n*)	22.1 (19)
Panlobular, % (*n*)	11.6 (10)
**Percent emphysema**	
Left upper lobe LAA, %	17.0 ± 11.7
Left lower lobe LAA, %	14.0 ± 11.1
Left lung LAA, %	15.8 ± 10.8
Right upper lobe LAA, %	16.5 ± 11.2
Right middle lobe LAA, %	17.3 ± 10.4
Right lower lobe LAA, %	13.1 ± 9.1
Right lung LAA, %	15.4 ± 9.0
Upper lung LAA, %	33.5 ± 22.1
Lower lung LAA, %	27.2 ± 18.8
Total lung LAA, %	15.6 ± 9.4
Emphysema severity, point	2.1 ± 0.5
**Air pollution**	
PM_2.5_, μg/m^3^	28.02 ± 3.38
NO_2_, ppb	18.20 ± 2.23
O_3_, ppb	24.44 ± 3.31

*BMI, body mass index; FEV_1_, forced expiratory volume in the first second; FVC, forced vital capacity; LAA, low attenuation area; mMRC, modified Medical Research Council*.

Based on HRCT scans, pulmonary emphysema was further classified into different subtypes (i.e., centrilobular, paraseptal, and panlobular emphysema). The percentage of predominant centrilobular, paraseptal, and panlobular emphysema were 66.3, 22.1, and 11.6%, respectively. The mean degree of emphysema in the total lung was 15.6 ± 9.4%. The mean emphysema severity was 2.1 ± 0.5 points.

### Air Pollution

One-year mean air pollution levels are depicted in Table 1. Levels of 1-year concentrations of PM_2.5_, NO_2_, and O_3_ to which study subjects were exposed were 28.02 ± 3.38 μg/m^3^, 18.20 ± 2.23 ppb, and 24.44 ± 3.31 ppb, respectively.

### Association of O_3_ With Predominant Panlobular Emphysema

The association of air pollution with emphysema subtypes is shown in Table 2. We observed that a 1 ppb increase in O_3_ was associated with 1.798-fold increased crude OR of panlobular subtype (95% CI: 1.073, 3.013; *p* < 0.05). After adjusting for age, sex, BMI, and smoking pack-years, 1 ppb increase in O_3_ was associated with 1.854-fold increased adjusted OR of panlobular subtype (95% CI: 1.069, 3.216; *p* < 0.05).

**Table 2:** Associations [odds ratio (OR)] of centrilobular and panlobular emphysema subtypes with paraseptal emphysema (reference) by 1-year average air pollution concentrations of PM_2.5_, NO_2_, and O_3_.

	**Crude OR (95% CI)**			**Adjusted OR (95% CI)**		
**Air pollution**	**Paraseptal**	**Centrilobular**	**Panlobular**	**Paraseptal**	**Centrilobular**	**Panlobular**
PM_2.5_, μg/m^3^	1	0.937 (0.799, 1.098)	1.151 (0.922, 1.436)	1	0.923 (0.784, 1.087)	1.110 (0.883, 1.395)
NO_2_, ppb	1	0.868 (0.683, 1.103)	0.870 (0.613, 1.236)	1	0.865 (0.680, 1.101)	0.850 (0.587, 1.232)
O_3_, ppb	1	0.933 (0.788, 1.106)	1.798 (1.073, 3.013)^*^	1	0.923 (0.774, 1.100)	1.854 (1.069, 3.216)^*^

*NO_2_, nitrogen dioxide; O_3_, ozone; PM_2.5_, particulate matter <2.5 μm in aerodynamic diameters*.

*Adjusted for age, sex, body mass index, and smoking pack-years*.

*Values in bold characters are deemed statistically significant*.

* *p < 0.05*.

### Associations of PM_2.5_, NO_2_, and O_3_ With BODE and Degree of Emphysema

One-year mean air pollution levels and the association of PM_2.5_, NO_2_, and O_3_ on BODE quartiles and percent emphysema are depicted in Table 3. Levels of 1-year concentrations of PM_2.5_, NO_2_, and O_3_ to which study subjects were exposed were 28.02 ± 3.38 μg/m^3^, 18.20 ± 2.23 ppb, and 24.44 ± 3.31 ppb, respectively. After adjusting for age, sex, BMI, and smoking pack-years, we observed significant associations between PM_2.5_ and left upper lobe LAA (β = 1.476), right upper lobe LAA (β = 1.296), left lower lobe LAA (β = 1.293), right middle lobe LAA (β = 1.202), right lower lobe LAA (β = 0.978), and emphysema severity (β = 0.059). Furthermore, we observed significant associations between PM_2.5_ and BODE quartiles, mMRC dyspnea scale, and exercise capacity scale.

**Table 3:** Associations between lung function, BODE quartiles, and percent emphysema (95% CI) with 1-year average air pollution concentrations of PM_2.5_, NO_2_, and O_3_.

**Air pollution**	**PM_**2.5**_**	**NO_**2**_**	**O_**3**_**
	**β coefficient (95% CI)**	**β coefficient (95% CI)**	**β coefficient (95% CI)**
**Lung function**			
FEV_1_, %	−0.724 (−1.976, 0.529)	−0.135 (−2.013, 1.742)	0.468 (−0.817, 1.753)
FEV_1_, L	−0.022 (−0.053, 0.010)	−0.003 (−0.050, 0.044)	−0.006 (−0.038, 0.027)
FEV_1_/FVC, %	−0.358 (−0.995, 0.278)	0.038 (−0.915, 0.992)	0.148 (−0.506, 0.801)
**BODE quartiles, point**	0.099 (0.024, 0.175)^*^	−0.066 (−0.182, 0.050)	0.054 (−0.027, 0.135)
BMI scale, point	0.003 (−0.021, 0.027)	−0.015 (−0.060, 0.031)	0.003 (−0.029, 0.034)
Airflow obstruction, point	0.024 (−0.052, 0.100)	−0.085 (−0.197, 0.026)	0.007 (−0.069, 0.083)
mMRC dyspnea, point	0.076 (0.023, 0.130)^*^	0.019 (−0.065, 0.102)	0.017 (−0.043, 0.077)
Exercise capacity, point	0.083 (0.017, 0.150)^*^	0.046 (−0.056, 0.148)	0.031 (−0.040, 0.103)
**Percent emphysema**			
Left upper lobe LAA, %	1.476 (0.813, 2.139)^*^	1.434 (0.387, 2.481)^*^	1.492 (0.813, 2.171)^*^
Left lower lobe LAA, %	1.293 (0.660, 1.927)^*^	0.922 (−0.086, 1.929)	0.866 (0.185, 1.546)^*^
Left lung LAA, %	1.414 (0.811, 2.018)^*^	1.236 (0.269, 2.203)^*^	1.206 (0.568, 1.845)^*^
Right upper lobe LAA, %	1.296 (0.665, 1.927)^*^	1.946 (1.008, 2.883)^*^	1.560 (0.939, 2.180)^*^
Right middle lobe LAA, %	1.202 (0.604, 1.800)^*^	0.687 (−0.269, 1.642)	1.126 (0.507, 1.746)^*^
Right lower lobe LAA, %	0.978 (0.481, 1.474)^*^	0.883 (0.106, 1.661)^*^	0.860 (0.342, 1.378)^*^
Right lung LAA, %	1.138 (0.640, 1.635)^*^	1.242 (0.462, 2.021)^*^	1.209 (0.705, 1.712)^*^
Upper lobe LAA, %	2.772 (1.535, 4.009)^*^	3.380 (1.473, 5.286)^*^	3.052 (1.812, 4.291)^*^
Lower lobe LAA, %	2.271 (1.238, 3.303)^*^	1.805 (0.154, 3.455)^*^	1.726 (0.621, 2.830)^*^
Total lung LAA, %	1.283 (0.769, 1.797)^*^	1.282 (0.461, 2.102)^*^	1.198 (0.662, 1.735)^*^
Emphysema severity, point	0.059 (0.029, 0.089)^*^	0.055 (0.008, 0.103)^*^	0.044 (0.012, 0.076)^*^

*BMI, body mass index; FEV_1_, forced expiratory volume in the first second; FVC, forced vital capacity; LAA, low attenuation area; mMRC, modified Medical Research Council; NO_2_, nitrogen dioxide; O_3_, ozone; PM_2.5_, particulate matter <2.5 μm in aerodynamic diameters*.

*Adjusted for age, sex, body mass index, and smoking pack-years*.

*Values in bold characters are deemed statistically significant*.

* *p < 0.05*.

Similarly, significant associations were observed between NO_2_ and right upper lobe LAA (β = 1.946), left upper lobe LAA (β = 1.434), right lower lobe LAA (β = 0.883), and emphysema severity (β = 0.055).

Meanwhile, we observed significant associations between O_3_ and right upper lobe LAA (β = 1.560), left upper lobe LAA (β = 1.492), right middle lobe LAA (β = 1.126), left lower lobe LAA (β = 0.866), right lower lobe LAA (β = 0.860), and emphysema severity (β = 0.044).

Furthermore, we observed that an increase in PM_2.5_, NO_2_, and O_3_ was associated with greater increases in upper lobe LAA than in lower lobe LAA.

## Discussion

We showed an association between O_3_ exposure and panlobular emphysema subtype. Importantly, air pollution (PM_2.5_, NO_2_, and O_3_) was associated with an increased degree of lobar emphysema, especially in the upper lobes. Our results suggest that particulate and gaseous pollution could have distinct impacts on lung lobes emphysema.

The annual PM_2.5_, NO_2_, and O_3_ levels in our study were following previous studies conducted in Taipei (33, 34). However, the mean PM_2.5_ levels in our study were nearly 3-fold higher than the WHO acceptable upper limit (annual PM_2.5_ of 10 μg/m^3^) (35). Meanwhile, the mean O_3_ levels in our study were lower than the acceptable upper limit of the United States EPA (annual O_3_ of 70 ppb) (36). Using paraseptal emphysema as the reference group, we found that exposure to O_3_ was associated with a higher OR of panlobular emphysema. O_3_ is formed by the photochemical dissociation of molecular oxygen into two oxygen atoms, followed by a combination between the oxygen atom and the molecular oxygen. Previous studies reported the role of apoptosis of alveolar epithelial cells and endothelial cells in the pathogenesis of emphysema (37–39). O_3_-induced oxidative stress could induce the activation of proteases (caspase-3) (40–42). Moreover, a previous *in vivo* study reported that exposure to 2.5 ppm O_3_ for 6 weeks resulted in alveolar enlargement and airway wall destruction associated with an increase in matrix metalloproteinase-12 (MMP-12) and caspase-3 (42). It was also reported that interleukin (IL)-13 may modulate O_3_-induced neutrophilic inflammation (43). Furthermore, IL-13 could promote alveolar macrophage elastase (MMP-12) upregulation, thus leading to emphysema (44–46). Moreover, previous studies reported that O_3_ was diffused in the alveolar periphery (47–49). It is also suggested that panlobular emphysema represents a more advanced phase of emphysema and COPD (14, 50). Together, this suggests that exposure to O_3_ can be associated with a higher risk of panlobular emphysema than paraseptal emphysema in patients with COPD. However, the association of O_3_ with panlobular emphysema warrants further investigations.

Next, we examined the association between air pollution and BODE quartiles and demonstrated that exposure to PM_2.5_ was associated with the increased BODE quartiles, the mMRC dyspnea scale, and the exercise capacity index. Our findings are consistent with previous studies showing that mMRC scores of patients with COPD when the air quality index (AQI) > 100 were higher than when AQI ≤ 100 (27). Moreover, exposure to diesel engine exhaust significantly decreased the exercise capacity compared to exposure to clean air in heart failure patients (26). The results indicate that exposure to air pollution increased the risk of COPD severity. Together, our data further suggested the adverse effects of exposure to PM_2.5_ to the BODE quartiles (i.e., BMI, airflow obstruction, dyspnea, and physical activities) in patients with COPD.

We identified air pollution (i.e., PM_2.5_, NO_2_, and O_3_) associated with increased percent emphysema of the total lung and emphysema severity. Previous studies showed that air pollutants may penetrate deeply into the lung and destroy the alveolar septa through the excessive reactive oxygen species (ROS) generation (51). It was reported that the ROS played an important role in the apoptosis of alveolar epithelial cells (52–55). ROS was necessary to activate the BCL2-associated X (Bax) protein, leading to cell death (53, 56, 57). Furthermore, the ROS can cause endothelial cell apoptosis (58, 59). Our results are consistent with previous studies (21, 22). Using 1-year average air pollution exposure, a previous study found that 5 μg/m^3^ increase in PM_2.5_ and 25 ppb increase in oxides of nitrogen (NO_*x*_) were associated with 0.6 (95% CI: 0.1, 1.2%) and 0.5 (95% CI: 0.1, 0.9%) increase in percent emphysema (21). A cohort study involving 19-year exposure to air pollutants demonstrated that 2 μg/m^3^ increase in PM_2.5_, 10 ppb increase in NO_*x*_, and 3 ppb increase in O_3_ resulted in 0.11 (95% CI: 0.03, 0.19), 0.06 (95% CI: 0.01, 0.12), and 0.13 (95% CI: 0.03, 0.24) increase in percent emphysema, respectively (22). In a study including 10-year exposure to O_3_, 5 ppb increase in O_3_ concentration was associated with increased emphysema severity (β = 0.94; 95% CI: 0.25, 1.64; *p* < 0.05) (60). However, the associations between air pollution and lobar emphysema are still unclear.

Due to the lung anatomy and aerodynamic motion of inhaled particles, we suspect that air pollution (especially particulate pollution) may have different impacts on the lung lobes. Our results further showed that air pollution was associated with lobar emphysema, especially in the upper lobes, by PM_2.5_, NO_2_, and O_3_. Our previous study found that a 1 μg/m^3^ increase in PM_2.5_ deposition in each lung lobe was associated with increases in %LAA (beta coefficient) of the same lung lobe (*p* < 0.05) (61). Because of particle physicochemical characteristics, lung geometric difference, and breathing pattern, the associations between air pollutants with lung lobe percent emphysema could be associated with our findings. In this study, we observed that an increase in PM_2.5_, NO_2_, and O_3_ was associated with greater increases in upper lobe LAA than in lower lobe LAA. This was consistent with a previous study showing that PM_2.5_ deposition was associated with higher emphysema severity in upper lobes than in lower lobes (61). This suggested that upper lobes might be preferentially impacted by air pollution than lower lobes. Furthermore, smoking-induced emphysema was also commonly observed in the upper lung lobes (13, 18). Smoking was also reported to be associated with centrilobular emphysema, which is mostly observed in upper lobe emphysema (14, 18, 20). The reasons for this upper lobe predominant distribution may include regional differences in lung physiology (i.e., ventilation/perfusion ratio, lymphatic flow, particle clearance, and intrapleural pressure) (13, 62). Although the ventilation predominated in the lower lung lobes, the ratio of ventilation to perfusion was higher in the upper lobes than in the lower lobes, which could favor the pathogenesis of upper lobe emphysema (63, 64). Meanwhile, the decrease in the lymphatic drainage in the upper lobes due to gravity could result in a decline in particle clearance, thus facilitating the development of emphysema (18, 63). Furthermore, the higher mechanical stress and more intrapleural pressures in the upper lung lobes may also result in the high distribution of emphysema in the upper lung lobes (65–67). However, this needs further investigations in future studies. Together, our data suggest that upper lung lobes could be more susceptible to impairment by air pollution.

The limitation of this study included its small sample size because the subjects recruited for this study depended on the number of admissions diagnosed with COPD during the study period. Furthermore, the lack of female representation in our study could be a limitation. The chemical components of PM_2.5_ (i.e., water-soluble ions, heavy metals, and polycyclic aromatic hydrocarbon) were not examined in our study. The effects of indoor pollution should also be clarified in future studies. We observed the associations between air pollution and the extent of emphysema, but the inflammatory responses and underlying mechanisms need to be investigated in the future. Because the sample size in our study was small, we did not adjust for previous pulmonary infections, alpha-1 antitrypsin levels, and socioeconomic status. These confounding factors should be included in future works. The subjects in our study were exposed to 1-year air pollution concentrations, which could be a limitation. Longitudinal analyses with long-term exposure to air pollution may better clarify these associations with percent emphysema.

## Conclusions

In conclusion, exposure to air pollution was associated with the degree and type of lobar emphysema in COPD. Our findings suggested that exposure to O_3_ was preferentially associated with panlobular emphysema than paraseptal emphysema in patients with COPD. Particulate and gaseous pollution may have distinct impact on the lung lobes. Moreover, this study showed an association between air pollution and the degree of emphysema, especially in the upper lobes. Air pollution could be associated with the severity of lobar emphysema in COPD.

## Data Availability Statement

The raw data supporting the conclusions of this article will be made available by the authors, without undue reservation.

## Ethics Statement

The studies involving human participants were reviewed and approved by the Ethics Committee of the Taipei Medical University-Joint Institutional Review Board approved this study (Approval No. N202003075). Written informed consent for participation was not required for this study in accordance with the national legislation and the institutional requirements.

## Author Contributions

H-CC planned the study and designed the experiments. NT and S-CH completed the manuscript. Y-HL, T-TC, K-YL, K-YC, S-MW, and H-YK completed the COPD data collection. C-DW completed the personal exposure assessment. HT, HD, and TT conducted the Multiple-Path Particle Dosimetry (MPPD) model. KC, H-PK, and Y-LL critically revised the manuscript. All authors analyzed and discussed the results, commented on the manuscript, and read and approved the final version of the manuscript for publication.

## Funding

This study was funded by the Ministry of Science and Technology of Taiwan (108-2314-B-038-093, 109-2314-B-038-093-MY3, and 108-2314-B-038-113-MY3).

## Conflict of Interest

The authors declare that the research was conducted in the absence of any commercial or financial relationships that could be construed as a potential conflict of interest.

## Publisher's Note

All claims expressed in this article are solely those of the authors and do not necessarily represent those of their affiliated organizations, or those of the publisher, the editors and the reviewers. Any product that may be evaluated in this article, or claim that may be made by its manufacturer, is not guaranteed or endorsed by the publisher.
