# Supplementary material for: Association Between Air Pollution and Lung Lobar Emphysema in COPD
Source: Front Med (Lausanne). 2021 Sep 21;8:705792. doi: 10.3389/fmed.2021.705792 (PMC8490678; doi:10.3389/fmed.2021.705792)
Supplement: Supplementary file 1 [file Table_1.DOCX]

**Association between air pollution and lung lobar emphysema in COPD**

Nguyen Thanh Tung^1,2†^ (MD.), Shu-Chuan Ho^3†^ (PhD.), Yueh-Hsun Lu^4,5^ (MD.), Tzu-Tao Chen^6^ (MD.), Kang-Yun Lee^6,7^ (MD., PhD.), Kuan-Yuan Chen^6^ (MD.), Chih-Da Wu^8,9^ (PhD.), Kian Fan Chung^10^ (MD., DSc.), Han-Pin Kuo^7^ (MD, PhD), Huynh Nguyen Xuan Thao^11^ (MD.), Hoang Ba Dung^2^ (MD.), Tran Phan Chung Thuy^12^ (MD., PhD.), Sheng-Ming Wu^6,7^ (PhD.), Hsiao-Yun Kou^3^ (MSc.), Yueh-Lun Lee^13^ (PhD.), Hsiao-Chi Chuang^3,6,14*^ (PhD.)

^1^International Ph.D. Program in Medicine, College of Medicine, Taipei Medical University, Taipei, Taiwan

^2^Otorhinolaryngology Department, Cho Ray Hospital, Ho Chi Minh City, Vietnam

^3^School of Respiratory Therapy, College of Medicine, Taipei Medical University, Taipei, Taiwan

^4^Department of Radiology, Shuang Ho Hospital, Taipei Medical University, New Taipei City, Taiwan

^5^Department of Radiology, School of Medicine, College of Medicine, Taipei Medical University, Taipei, Taiwan

^6^Division of Pulmonary Medicine, Department of Internal Medicine, Shuang Ho Hospital, Taipei Medical University, New Taipei City, Taiwan

^7^Division of Pulmonary Medicine, Department of Internal Medicine, School of Medicine, College of Medicine, Taipei Medical University, Taipei, Taiwan

^8^Department of Geomatics, National Cheng Kung University, Tainan, Taiwan

^9^National Institute of Environmental Health Sciences, National Health Research Institutes, Miaoli, Taiwan

^10^National Heart and Lung Institute, Imperial College London, London, UK

^11^Ho Chi Minh City University of Medicine and Pharmacy, Ho Chi Minh City, Vietnam

^12^Otorhinolaryngology Department, Faculty of Medicine, Vietnam National University Ho Chi Minh City, Ho Chi Minh City, Vietnam

^13^Department of Microbiology and Immunology, School of Medicine, College of Medicine, Taipei Medical University, Taipei, Taiwan

^14^Cell Physiology and Molecular Image Research Center, Wan Fang Hospital, Taipei Medical University, Taipei, Taiwan

**Table S1**. Variables and point value used for the calculation of body mass index scale, airflow obstruction index, MMRC dyspnea scale, and exercise capacity (BODE) index.

|  | **Variable** | **Points on BODE Index** | | | |
| --- | --- | --- | --- | --- | --- |
|  |  | **0** | **1** | **2** | **3** |
| Body-mass index scale | Body-mass index, kg/m^2^ | >21 | ≤21 |  |  |
| Airflow obstruction index | FEV1, % of predicted | ≥65 | 50-64 | 36-49 | ≤35 |
| mMRC dyspnea scale |  | 0-1 | 2 | 3 | 4 |
| Exercise capacity index | Distance walked in 6 min, m | ≥350 | 250-349 | 150-249 | ≤149 |

FEV_1_: forced expiratory volume in the first second; mMRC: Modified Medical Research Council
